# Evaluation of the degree of mycophilia-mycophobia among highland and lowland inhabitants from Chiapas, Mexico

**DOI:** 10.1186/1746-4269-9-36

**Published:** 2013-05-26

**Authors:** Felipe Ruan-Soto, Javier Caballero, Carlos Martorell, Joaquín Cifuentes, Alma Rosa González-Esquinca, Roberto Garibay-Orijel

**Affiliations:** 1 Facultad de Ciencias Biológicas, Universidad de Ciencias y Artes de Chiapas, Tuxtla Gutiérrez, Mexico; 2 Instituto de Biología, Universidad Nacional Autónoma de México, México D.F., Mexico; 3 Facultad de Ciencias, Universidad Nacional Autónoma de México, México D.F., Mexico

**Keywords:** Ethnomycology, Ethnobiology, Local mycological knowledge, Edible mushrooms, Mycophilia-mycophobia

## Abstract

**Background:**

Mushrooms generate strong and contrasting feelings ranging from extreme aversion to intense liking. To categorize these attitudes, Wasson and Wasson coined the dichotomic terms “mycophilia” and “mycophobia” in 1957. In Mesoamerica these categories have been associated to ecological regions. Highland peoples are viewed as mycophiles, whereas lowland inhabitants are considered mycophobes. However, this division is based on little empirical evidence and few indicators. This study questioned whether mycophilia and mycophobia are indeed related to ecological regions through the evaluation of 19 indicators tested in the highlands and lowlands of Chiapas, Mexico.

**Methods:**

The heterogeneity of attitudes toward mushrooms was explored in terms of ecological region and sociocultural variables. Information was obtained through structured interviews in 10 communities in Los Altos de Chiapas (highlands) and the Selva Lacandona (lowlands). We analyzed indicators separately through *χ*^2^ tests and multivariate techniques. The Mycophilia-Mycophobia Index was also used in the analysis. To assess which factors better explain the distribution of attitudes, we built 11 models using the Beta probability-density function and compared them with the Akaike Information Criterion.

**Results:**

Most people had positive attitudes in both ecological regions. The classification and ordination analyses found two large groups comprising both highland and lowland towns. Contrary to expectation if mycophilia and mycophobia were mutually exclusive, all the fitted probability distributions were bell-shaped; indicating these attitudes behave as a continuous variable. The model best supported by data included occupation and ethnicity. Indigenous peasants had the highest degree of mycophilia.

**Discussion:**

Results suggest the studied populations tend to be mycophilic and that their attitudes are not dichotomic, but rather a gradient. Most people occupied intermediate degrees of mycophilia. Despite the remarkable similarity in the degree of mycophilia between ecological regions, the Principle-Coordinates Analysis shows differences in the specific way in which people from either region establishes a cultural relationship with mushrooms. The comparison of models suggests that sociocultural variables explains the differences better than ecological regions do. The obtained results are evidence of mycophilia among lowlands inhabitants in the Mayan region and of the fact that the mycophilia-mycophobia phenomenon is not expressed as a bimodal frequency distribution.

## Abstract (es)

**Resumen:**

**Introducción**

Los hongos son capaces de generar sentimientos y emociones fuertes y contrastantes: aversiones extremas o aficiones intensas. Para categorizar a estas actitudes, Wasson y Wasson propusieron en 1957 los conceptos totalizadores y dicotómicos de micofilia y micofobia. En Mesoamérica esta separación se conceptualizó en función del piso ecológico, considerando a pueblos de tierras altas como micófilos y a los de tierras bajas como micófobos. Sin embargo, esta clasificación se ha realizado con base en escasa evidencia empírica y evaluando muy pocos indicadores. El presente estudio trata de probar la hipótesis de si las actitudes de micofilia y micofobia están relacionadas con el piso ecológico en que habitan las personas, a través de la evaluación de 19 indicadores en tierras altas y tierras bajas de Chiapas, México.

**Método**

Se exploró cómo se comporta la población y la heterogeneidad en sus actitudes hacia los hongos, así como el efecto del piso ecológico y variables socioculturales. Se analizaron los indicadores de manera separada a través de pruebas de *χ*^2^ y de técnicas multivariadas. Se propone el uso del Índice de Micofilia-Micofobia. Para evaluar qué factores explican mejor la distribución de las diferentes actitudes se construyeron 11 modelos usando la función de densidad de la probabilidad Beta y se compararon a través del Criterio de Información de Akaike.

**Resultados**

La mayoría de las personas tienen actitudes positivas en ambos pisos ecológicos. Los análisis de clasificación y ordenación mostraron dos grupos que incluyen poblados de ambos pisos, al contrario del Análisis de Coordenadas Principales que muestra una separación por piso ecológico. Contrario a lo esperado, el fenómeno de micofilia-micofobia no resulto ser mutuamente excluyente sino se ajustó a una distribución de probabilidad acampanada, es decir, mostrando a las actitudes como una variable continua. El modelo más robusto incluye la ocupación y la condición étnica siendo los campesinos indígenas los que tienen el máximo grado de micofilia.

**Discusión**

Los resultados sugieren que los pueblos estudiados tienden hacia la micofilia y no presentan una distribución de frecuencias dicotómica. Por el contrario, la mayoría de las personas tienen un grado intermedio de micofilia. Sin embargo, el análisis de Coordenadas Principales muestra que existen diferencias en la manera específica en que los habitantes de cada piso ecológico se relacionan con los hongos. La evidencia muestra que la micofilia es generalizada entre los pueblos mayas de tierras bajas y que el fenómeno de micofilia-micofobia no se expresa como una distribución de frecuencias bimodal.

## Background

Some Aspects of relationships between humans and mushrooms such as mycological knowledge and mushroom management as well as attitudes are a product of how, when, and in what measure cultures construct their notion of these organisms given their particular circumstances [1]. That is, those relationships are a product of an eminently historical process, both natural and social.

Mushrooms, unlike most organisms, generate strong and contrasting feelings in people [2]. They can provoke extreme aversions as well as intense liking and joy. These positive or negative feelings are not generally rationalized because they are part of the culture of a given social group. This phenomenon was first tackled in the mid-twentieth century [3]. To characterize the diverging ways in which entire societies approach mushrooms, Wasson and Wasson [4] proposed the generalizing, dichotomic, and mutually exclusive terms mycophilia and mycophobia. Mycophilia refers to peoples who like and appreciate mushrooms and mycophobia to peoples who feel aversion toward these organisms.

With time, more complete definitions of these concepts have been constructed. Mycophilic people display special interest toward mushrooms, which are part of their diet, their traditional medicine, and other purposes such as religious ceremonies and healing practices. On the other hand, mycophobic people have aversion toward mushrooms, an attitude of contempt or even fear to them. They try not to touch them, perceive them as something associated to rotting, have no traditional names for different species of mushrooms, and even have sayings and refrains to enforce negative attitudes to mushrooms; they cannot identify the species in their territory and, evidently, they do not consume them [3,5,6].

Fericgla [5] characterized different European peoples as eminently mycophilic (e.g. Catalans or Russians) or clearly mycophobic (e.g. Castilian or Valencian). This exercise was replicated characterizing other regions and peoples of Asia, the South Pacific and the Americas [5,7-9]. For the Mesoamerican and Amazonian regions, mycophilia and mycophobia have been described to be associated to ecological regions: peoples from the highlands were characterized as mycophilic, while peoples from the lowlands were described as mycophobic [6,10-12]. In general, this classification has been done based on general perceptions or on the number of recognized and used species, but without clear, systematic or standardized criteria.

Mapes et al. [12] categorized Mesoamerican and Amazonian peoples based on four indicators: 1) number of mushroom species used as food, 2) diversified use, 3) commerce and 4) mycolatry (fungi worship). They conclude that highland Mesoamerican peoples are mycophiles, whereas lowland peoples are mycophobes. They suggest that when pre-Columbian Maya migrated from the highlands to the lowlands, they experienced a process of land appropriation in which plants –a more abundant resource in rainforests– took the cultural niche that mushrooms formerly occupied. For both Mapes et al. [12] and this study, the highlands are tropical regions above 1500 m.a.s.l., with a vegetation of temperate forests including *Pinus, Quercus,* and/or *Liquidambar*, subject to the influence of frost during winter. On the other hand, the lowlands are understood to be lands below 1000 m.a.s.l., with evergreen or sub-evergreen rainforest, and without frost influence.

Recently, practices of formerly unstudied peoples from tropical lowlands and other highland regions have been documented, leading to a reconsideration of the current theory on mycophilia as a function of ecological zone. These works show that not all peoples from the highlands approach mushrooms in a similar way [13-15] and that most lowland peoples are not mycophobes [16-19]. Furthermore, Arora and Shepard [2] point out that ethnomycological works developed in recent years document a wider and more diverse range of cultural attitudes toward mushrooms, possibly shaped by cultural and ecological aspects. Thus, if attitudes toward mushrooms are effectively expressed through a spectrum of actions and conceptions, any evaluation should consider as many of the aspects that make mushrooms culturally important as possible.

If this is so, mycophilia and mycophobia may be understood differently – not as mutually exclusive attitudes that a whole cultural group has (as originally posed by Wasson [3]) –, but as a gradient on which societies can be considered more or less mycophilic-mycophobic. In this way, the attitude of a population toward mushrooms could be expressed as a frequency distribution tending to one extreme or the other.

**Figure 1:**
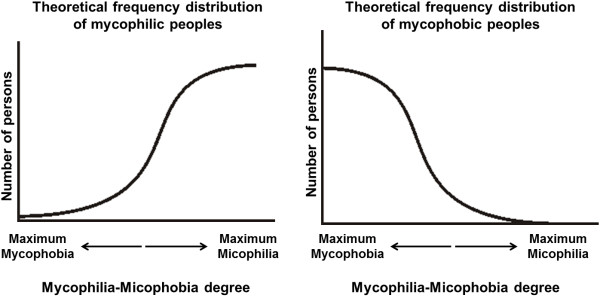
**Theorical frequencies distribution of a mycophile and a mycophobe people according to Fericgla **[5]**.**

Thus, a mycophilic and a mycophobic people, as described by Fericgla (1994), could have a theoretical frequencies distribution such as those observed in Figure 1. It would be expected that the frequencies distribution of the highlands towns had a topology similar to the left side of Figure 1, while the frequencies distribution for the lowlands towns would resemble its right side. That is, according to available literature, the whole population (highlands and lowlands) should have a bimodal distribution.

Although ethnomycology as a discipline emerged with the analysis of this dichotomy [2], there are still many questions to be answered: Are mycophilic and mycophobic attitudes mutually exclusive, or are there continuous degrees between them? Are inhabitants of lowlands indeed more mycophobic than highland peoples? Are ecological regions a factor that explains differential attitudes toward mushrooms? Are there other factors that influence these different attitudes?

Our objective was to quantitatively evaluate the degree of mycophilia-mycophobia in populations from highlands and lowlands. With this, we intended to test the hypothesis that attitudes of mycophilia-mycophobia are related to the inhabited ecological region, as well as to explore the nature of the attitudes of people toward mushrooms. We further explore the role some sociocultural variables, such as ethnicity, occupation, and gender have in these contrasting attitudes toward mushrooms [20-22].

## Methods

### Study area

**Figure 2:**
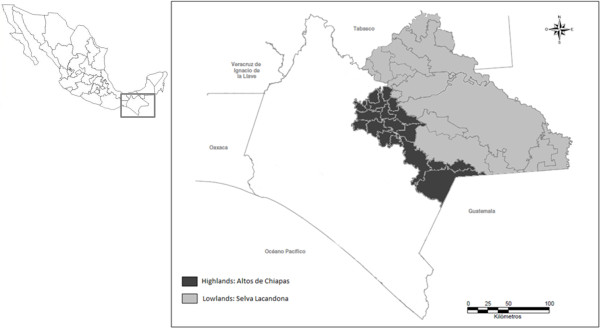
**Study area: Chiapas Highlands and the Lacandon rainforest, Chiapas, Mexico.** Map design by Andres Cruz Solis (YAXAL-NA Consultancy, Mexico).

Fieldwork was carried out in two regions of the State of Chiapas, Mexico: Los Altos de Chiapas (Highlands) and Lacandon Rainforest (Lowlands) (Figure 2). The Lacandon rainforest is a region with altitudes ranging between 0 and 1200 m a.s.l., with a warm humid climate and evergreen or sub-evergreen rainforest [23]. Due to human activity, the original vegetation has been transformed to grasslands and “acahuales” in different succession degrees. The region is integrated by 14 municipalities with a total population of 713,944 [24]. In this region there are three native indigenous groups: Lacandon, Ch’ol and Tseltal, as well as diverse mestizo groups and migrant indigenous groups mainly represented by Tseltal from the highlands and Mam. Indigenous population represents 62% of the total population. Men and women have a balanced proportion approaching 50% [24].

Chiapas Highlands is a mountainous region with altitudes between 1200 and 2700 m a.s.l. It has a temperate climate and a vegetation of pine-oak, pine-oak-liquidambar, and cloud forest, as well as large plantation areas [25]. The region includes 19 municipalities with a total population of 671,170. 49% of the indigenous populations are speakers of Tsotsil, Tseltal, Tojolabal, and Chuj. The proportion of men and women is balanced [24].

In all Chiapas, around 20 000 species of mushrooms are estimated to be present; only 2% of them have been registered [16]. There are no studies documenting the richness of mushrooms in each ecological region in detail, however the richness of the highlands is presumed to outstrip that of the lowlands in a 3 to 1 proportion (Cifuentes com. pers.). Furthermore, in the highlands ectomycorrhizal mushrooms with large and fleshy fruit bodies are more common while in the lowlands, smaller, leathery, saprobial mushrooms are more frequent. Different studies have demonstrated the great quantity of recognized and used species and explored how these organisms fit into peoples’ worldview, the naming and classification of species, the ethnomycological knowledge they have built around them, and the uses they give them [15,16]. There are cognitive similitudes registered among inhabitants of both regions: the logic behind the naming and classification of mushrooms, the knowledge about their biology and ecology, and their usefulness (edible, medicinal, ludic, ornamental, and recreational). Notwithstanding such similarities, there are marked differences among ecological regions, such as the number of species consumed −24 in the highlands and only 11 in the lowlands–. With regard to toxic species, there is no systematic study recording their identity or number in each ecological region; however, it seems all the species considered as deathly have been registered exclusively in the highlands due to the mycorrhizal association between them and *Pinus* spp. and *Quercus* spp.

### Data collection and analyses

To evaluate the degree of mycophilia-mycophobia in the study area, a method for gathering and analyzing information was designed. Based on literature and previous fieldwork, nine important cultural domains describing the attitude of a person toward mushrooms were identified: 1) knowledge and use of edible species [3,16,26-30]; 2) knowledge of toxic species [19,31]; 3) knowledge of species without cultural importance [5]; 4) worldview or “Imago mundi”; that is, the way people explains their universe, its origin and order, and how humans participate in that order [19,32]; 5) multiple use of mushroom species, not only edible [33]; 6) presence of specialists in mushroom picking [34]; 7) ethnoecological knowledge [18,32,35]; 8) transmission of ethnomycological knowledge [36]; and 9) perceived importance of mushrooms as a group of organisms [5]. Through these domains, 19 indicators were selected to describe the general attitude of a person toward mushrooms (Table 1). A structured interview including one question per indicator plus sociocultural information (ethnicity, occupation, origin, community and ecological region) was constructed (see Additional file 1). Answers were codified with a value of 1 when the answer was equivalent to a positive attitude, 0.5 when it corresponded to a neutral attitude and 0 when the answer was equivalent to a negative attitude. The Mycophilia-Mycophobia Index (MMI) value was calculated by adding the score obtained for each indicator, so that each interviewee had a MMI value between 0 (mycophobia maximum) and 19 (mycophilia maximum).

**Table 1:** Cultural domains and indicators used in the interview and for the Mycophilia-Mycophobia

**Cultural domains**	**Indicators**
1. Knowledge and use of edible species	1.1. Recognition of edible species
	1.2. Taxonomic knowledge of edible species
	1.3. Harvest
	1.4. Consumption of edible species
	1.5. Alimentary appreciation
	1.6. Special food consideration
	1.7. Culinary knowledge
	1.8. Attitude toward edible species
2. Knowledge of toxic species	2.1. Recognition of toxic species
	2.2. Morphological knowledge of toxic species
3. Knowledge of species without cultural significance	3.1. Attitude toward species without cultural significance
4. Worldview	4.1. Existence of tales or myths of origin including mushrooms
5. Multiple use	5.1. Presence of non-alimentary uses
6. Specialists	6.1. Presence of mushroom harvest and/or salespeople
7. Ethnoecological knowledge	7.1. Knowledge of the role of mushrooms in ecosystems
	7.2. Knowledge of the relation between mushrooms and animals
8. Ethnomycological knowledge transmission	8.1. Presence of knowledge transmission mechanisms
9. Importance of mushrooms as a whole	9.1. Attitude toward mushrooms as a whole
	9.2. Perceived importance of mushrooms as a whole

Fieldwork was designed to record the variation between highlands and lowlands. Random samples of the people were obtained from different towns. Ten communities were selected in each ecological region according to their size, ethnical groups, and dialectal variants (Table 1). In each town, individuals over 15 years of age were interviewed. Interviewees were chosen in a random way using maps of housing for each community, totaling 115 interviewees in the lowlands and 106 in the highlands (221 in both ecological regions) (Table 2). Non-structured and semi structured interviews were also carried out [37] to clarify the context of the local reality and the ideas expressed by the interviewees. All interviews –structured, non-structured and semi structured –were carried out in the houses of the interviewed, having previously agreed on a convenient time for them. Interviewers were careful not to let visiting times interfere with a random selection of the sample. Responses to the interviews were written down; for structured interviews pre-established formats were used (see Additional file 1) and the rest of the interviews were registered in field diaries.

**Table 2:** Communities where the interviews were conducted

**Highlands: Chiapas Highlands**			**Lowlands: Lacandon rainforest**		
**Community**	**Ethnic affiliation**	***n***	**Community**	**Ethnic affiliation**	***n***
Chamula	Tsotsil	13	Naha	Lacandon	13
Zinacantan	Tsotsil	10	Lacanja-Chansayab	Lacandon	13
Bazom	Tsotsil	11	Agua Azul	Tseltal, Mestizo and migrant indigenous people	13
Amatenango del Valle	Tseltal	10	Masanilha	Tseltal	10
Tenejapa	Tseltal	10	Frontera Corozal	Cho´l and Mestizo	10
Tziscao	Chuj and Mestizo	11	Las Nubes	Tseltal	10
Antela	Mestizo	10	Flor de Marques	Mestizo and migrant indigenous people.	15
San Antonio Lindavista	Tojolabal and Mestizo	10	Reforma Agraria	Mestizo ad migrant indigenous people.	10
Teopisca	Mestizo and Tseltal	11	Playon de la Gloria	Mestizo and migrant indigenous people.	11
San Cristobal de Las Casas	Mestizo and Tsotsil	10	Palenque	Mestizo and Cho´l	10

To analyze the relation between ecological region, sociocultural factors, and positive, neutral, or negative attitudes, we followed two procedures. First, we analyzed the 19 indicators separately through χ^2^ tests to determine which indicators presented significant differences between ecological regions. Second, to explore whether populations form groups based on ecological region or other sociocultural variables, multivariate techniques were used. Each indicator was a trait to evaluate (qualitative, three states) and an average of interviewees was calculated to obtain a relative value of each indicator per community. A distance matrix was calculated using the method of average taxonomic distance. With these values a Cluster Analysis by Complete Linkage method and a Principal Coordinates Analysis (PCO) were carried out in NTSYS (Numerical Taxonomy and Multivariate Analysis System) ver. 2.11x for PC [38].

To evaluate which ecological and sociocultural factors better explain the distribution of different attitudes toward mushrooms in the human population we built several models using the beta probability-density function [39]. This distribution was chosen because the attitude towards mushrooms may be either a dichotomic (mycophiles-mycophobes) or gradual variable. The beta distribution is extremely flexible, and may assume a wide range of shapes, from an extremely bimodal form with two peaks at extreme MMI values (as it would be expected if mycophilia and mycophobia were mutually exclusive) to one single bell-shaped curve in which most members of a population have intermediate MMI values (as would be expected if the attitude towards mushrooms were a continuous variable). Each model consisted of one or more beta distributions that described the probability density of observing an individual with a given MMI in a population within an ecological region or having certain sociocultural attribute. In total, we produced eleven models by fitting through maximum likelihood a beta distribution to different subsets of the MMI values sampled: a) Null model: The probability of sampling a person with any given MMI value is independent of the ecological region and sociocultural variables; b) Single-factor models: In these, different probability distributions were fitted to data according to either ecological region (highlands-lowlands), occupation (peasant-non peasant: where peasants are defined as people whose occupations put them in direct contact or usage of the natural spaces, such as those in land cultivation, stockbreeding, extraction of forest resources, forest rangers, or people involved in the development of productive projects in rural communities. Non-peasants are defined as people whose occupation does not require such contact, like people working in commerce, transportation, and public service, to name a few), ethnicity (indigenous-mestizo: where indigenous are defined as those who recognize themselves as such and speak an indigenous language and mestizos are defined as those speaking Spanish as a first language), gender (man-woman), or origin (native-migrant); c) Two-factor models with ecological region: In all of these models, the probability distribution of MMI was assumed to depend on the ecological region and one of the sociocultural factors (e.g., ecological region and gender, ecological region and ethnicity, etc.); and d) Two-factor sociocultural model: This model included the joint effect of occupation and ethnicity on MMI, and was chosen because there is a vast amount of literature that points out that these two factors are the most important ones in determining the relationship between people and natural resources [21].

The 11 models were then compared with the Akaike Information Criterion (AIC) [40]. This procedure allows for organizing the hypotheses (expressed as models) into a hierarchy that formally indicates the evidence supporting each one, allowing one to select the best among the competing models. If any model has an AIC value two units lower than another*,* it is concluded that the former is better supported by the data. If the difference in AIC values is smaller than two units, both models have similar support and it is impossible to select one over the other [40].

## Results

### Frequency distribution for the 19 indicators in the highlands and lowlands

**Figure 3:**
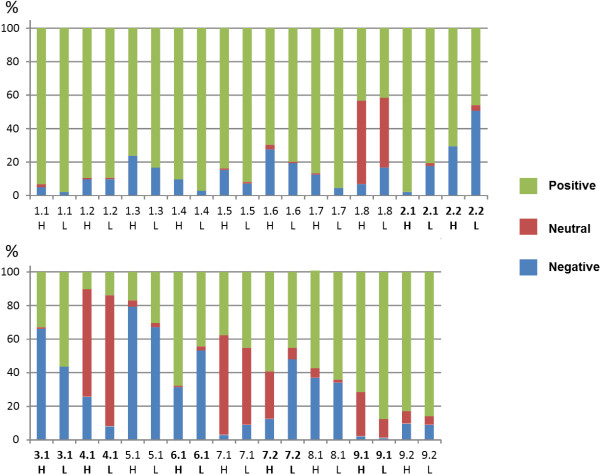
**Frequency distribution of the 19 indicators grouped in the nine cultural domains.** Symbology: 1.1. = Indicator number (See Table 1 for the description of each item), H = Highlands, L = Lowlands. In bold letters are the significantly different indicators between highlands and lowlands (χ^2^ test, p < 0.05, d.f. = 2).

There was a significantly greater frequency (χ^2^ tests, *p* < 0.05) of positive attitudes in the highlands regarding recognition and morphology of toxic species (items 2.1 and 2.2 in the structured interview) and presence of specialists (6.1). In contrast, lowlands showed significantly more positive attitudes towards species without cultural significance (3.1) and mushrooms as a group (9.1), as well as more frequent neutral or negative attitudes in terms of myths (4.1) and ethnoecological knowledge of the relation between mushrooms and animals (7.2). No significant differences were found for the 12 indicators related to knowledge and use of edible mushrooms, knowledge of mushroom’s role in the ecosystem, knowledge transmission, percieved importance of mushrooms, and the existence of non-alimentary uses (Figure 3).

Except for the indicator related to attitude toward species without cultural significance (3.1) in the highlands, and the one related to presence of non-alimentary use (5.1) for both ecological regions, indicators in both ecological regions show that most people have positive attitudes and knowledge (Figure 3) of mushrooms.

### Ordination and classification of communities according to their attitudes toward mushrooms

**Figure 4:**
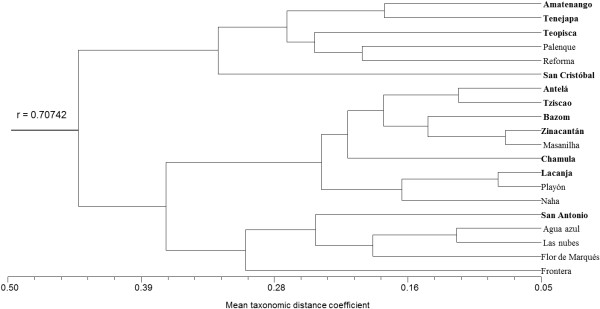
**Cluster analysis for communities by the complete linkage method.** In bold are the communities from the highlands.

**Figure 5:**
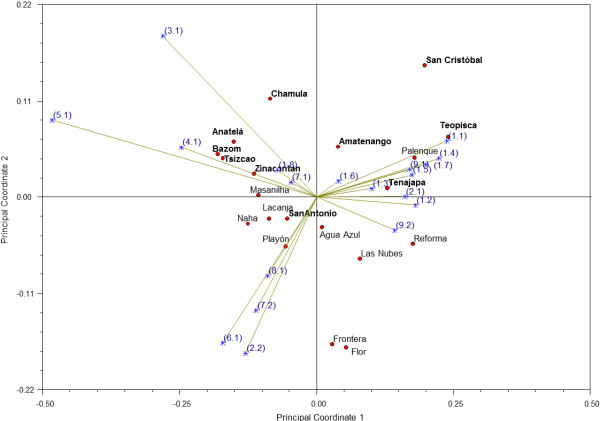
**Principal coordinate analysis by communities.** In bold letters are the communities from the highlands. Between parentheses are the indicators (See Table 1 for the description of each item).

The classification analysis found two large groups, each comprising both highland and lowland communities (Figure 4). The PCO analysis suggests the apparent formation of two groups. With the exception of Palenque and San Antonio, the highland communities do not mix with the lowlands (Figure 5). The most important indicators were items 5.1, 4.1, and 3.1 of the interview, that is, in the communities on the upper left quadrant of the graph there were more people aware of non-alimentary uses and tales and myths including mushrooms, and with a positive attitude towards species without cultural significance. However, positive values along the second principal coordinate axis correspond to communities mainly from the highlands that were characterized by a greater fear of species without cultural significance. Lowland communities with negative second PCO values included more people without knowledge of the morphology of toxic species or the relationships between animals and mushrooms, and which have less mushroom specialists in their communities.

### Probability distributions of the Mycophilia-Mycophobia Index (MMI)

**Figure 6:**
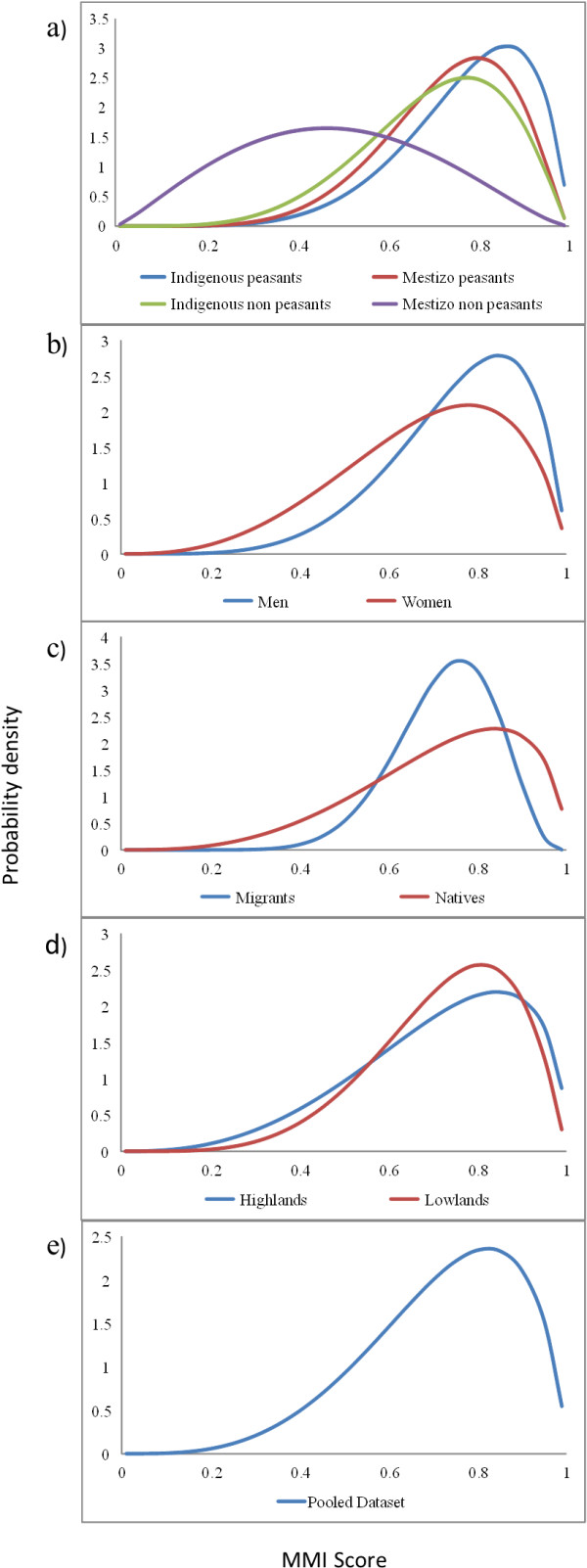
**Probability density distribution of the mycophilia-mycophobia index (MMI).** Models that include different sociocultural and ecological factors: **a**) Two-factor sociocultural model (occupation-ethnicity) **b**) Single-factor models: gender (man-woman), **c**) Single-factor models: origin (native-migrant), **d**) Single-factor models: ecological region (highlands-lowlands), and **e**) Null model: (pooled dataset). MMI = Micophilia-Micophobia Index.

Contrary to what would be expected if mycophilia-micophobia were mutually exclusive, all the fitted probability distributions were bell-shaped. This means that that there is a greater probability of finding people with intermediate degrees of mycophilia-mycophobia than extreme mycophobes or mycophiles. However, an overall trend towards moderate to high mycophilia was observed in the entire population (Figure 6e). The model that was best supported by the data included occupation and ethnicity, while the remaining models would be discarded as they all have much lower AIC values (Table 3). Indigenous peasants had the highest degree of mycophilia, followed by mestizo peasants and indigenous non-peasants, which had similar attitudes among themselves. Mestizo non-peasants had the lowest degree of mycophilia in the studied population (Figure 6a).

**Table 3:** Akaike Information Criterion (AIC) values for the compared models

**Model**	**AIC**
Occupation-Ethnicity	−235.5
Occupation	−218.1
Ecological region-Ethnicity	−214.3
Ecological region-Occupation	−206.1
Ethnicity	−205.5
Origin	−202.4
Gender	−201.9
Ecological region-Origin	−199.2
Ecological region-Gender	−198.8
Null model	−193.8
Ecological region	−193.7

All the models that accounted for the remaining sociocultural factors had a greater support than the null model, suggesting that gender and origin also determine the degree of mycophilia (Table 3). Men and natives were more mycophilic than women and migrants, respectively (Figure 6b-c).

Models in which ecological region and sociocultural variables interacted received the same or less support than the models with the respective sociocultural variables alone. Also, the null model had a similar support as the model with ecological region, making them indiscernible (Table 3). The MMI values in highlands and lowlands are quite similar, and thus resemble the distribution fitted for the null model (Figure 6d-e). Thus, all evidence points to sociocultural differences among populations (particularly regarding occupation and ethnicity) as better explanatory factors of mycophilia-mycophobia than ecological region.

## Discussion

With the development of ethnomycological studies in lowlands around the world in the last decade, mycophobia is no longer considered a general pattern [16]. With the results obtained from the 19 indicators, there is no evidence supporting the existence of communities or cultural groups completely mycophilic or mycophobic in the highlands or lowlands of Chiapas. Our evidence shows that all cultural groups have members with positive and negative attitudes toward mushrooms. Evaluating and quantifying these attitudes as a group can place people along a gradient of mycophilia. Furthermore, the degree of affinity or aversion towards mushrooms does not depend on the ecological region people inhabit, although the specific form in which mycophilia manifests differs between highland and lowland populations. The observed differences in mycophilia seem to be best explained by sociocultural factors such as gender, origin, and, more importantly, occupation and ethnicity.

According to the results of Fericgla [5], Mapes et al. [12], and other authors [6,11], a contrast between ecological regions was expected. This would appear in the lowlands as a greater frequency of negative attitudes, i.e., a biased distribution of MMI towards low values. However, this was not the case as most lowland inhabitants seem to be more mycophilic than mycophobic, and their distribution of MMI values was quite similar to that observed in the highlands. For almost every indicator, the frequency of positive or neutral attitudes is greater than that of negative ones regardless of the ecological region. These results are in line with recent findings that lowland people are not mycophobes [16]. Further research, such as was presented here, is required to assess whether the ecological-region model for explaining the degree of mycophilia of peoples still holds for other tropical regions of the world.

A difference between both regions would also be seen as a clear separation of highland and lowland populations in both the phenogram and the PCO. However, the results do not support this. The expected contrast in responses by ecological region was not observed at all in the phenogram, and only over the second (and thus less explicative) axis in the PCO. Only for some indicators was there a significant difference between ecological regions. Among these, three are noteworthy: in the highlands, most people (70%) know how to recognize poisonous mushrooms (30% in lowlands), there are more people with a negative attitude toward species without cultural importance (66% in highlands; 33% in lowlands), and there are more people (25%) who know tales which indicate negative attitudes toward mushrooms than in the lowlands (7%). In the highlands, people recognize poisonous species based on their shape, color, unpleasant odor, bitter taste, substrate, or type of vegetation where they are found. In the lowlands, contrastingly, people explain that they “only learn to recognize the ones that can be eaten, not those that cannot”. This is, the knowledge transmission is focused on people learning the characteristics of used mushrooms, while little attention is paid to poisonous or unused mushrooms. This may be based in the apparent absence of deathly species in the lowlands. However, it does not mean that there are no deathly species in this ecological region, they simply have not been formally studied. This mechanism has been observed in many European peoples [5], but it is a topic that has been overlooked by Latin American ethnomycology. Also, in the highlands people expressed a greater fear of touching unrecognized species, thinking that they could be harmed by these. In this region it is common to find phrases like “It’s dangerous to eat mushrooms… if you eat them you could die”. Such expressions might have an origin in campaigns the health authorities of the state of Chiapas launched following deadly cases of wild mushroom intoxication. The consumption of wild mushroom species was discouraged through messages broadcast on various media indicating the dangers involved in this activity [41]. While this campaign is fairly recent (2005 on), its impact on the perception toward this resource in the highlands of Chiapas can already be appreciated.

Thus, it seems that despite the remarkable similarity in the degree of mycophylia observed between ecological regions, there are differences in the specific way in which people from both regions establish their cultural relationship with mushrooms. People in the highlands show a more fearful and cautious attitude towards mushrooms, but they have developed strategies that allow them to exploit this resource intensively. These include a corpus of knowledge on poisonous species which is transmited among people, and the presence of trustworthy specialists who can accurately identify edible species. Contrastingly, people from the lowlands have limited knowledge regarding toxic species and do not recognize specialists in the identification of mushrooms. However, they also have less negative attitudes toward culturally unimportant mushrooms. This qualitative difference is clear in the second PCO axis, in which lowland and highland towns are sharply differentiated. This is consistent with the literature discribing Latin American highlands [28] and lowlands [16].

The comparison of models through AIC values clearly indicates that the model best supported by the data included occupation and ethnicity (Table 3). This model puts indigenous peasants at one extreme, as the group with the greatest degree of mycophilia, and Mestizo non-peasants at the other, as the group with the lowest degree of mycophilia (Figure 6a). Peasants live in direct contact with the elements of nature, where resources from the wild are used every day [42]. Furthermore, several works have pointed out the profound knowledge and management techniques that indigenous Mesoamerican groups have [21,43]. The indigenous-peasant group brings together the above mentioned heritage of a vast traditional ecological knowledge and greater direct dependence on the environment. On the other hand, the mestizo non-peasant group is quite opposite, with global knowledge and a scarce proximity-environmental dependence. Furthermore, by living in urban areas, or having more frequent contact with them, mestizo non-peasants are exposed to health department campaigns that point out the dangers of wild mushrooms. Consequently, this population is more susceptible to stop using, and even fearing these organisms.

The ordination analyses (Figures 4 and 5) also showed that differences in attitudes toward mushrooms depend on occupation and ethnicity. Communities from the first group in the classification analysis (Palenque, San Cristobal de Las Casas, Amatenango, Tenejapa, Teopisca, and Reforma) share sociodemographic characteristics: the first four are among the six communities with a greater proportion of non-peasant population. While Teopisca has a low percentage of non-peasants, it is one of the communities with a greater proportion of mestizos (as are Palenque and San Cristobal). On the other hand, San Cristobal de Las Casas, Teopisca, and Palenque are the communities with the greatest degree of urbanization. The PCO supports this grouping. Communities from the second group, such as Playon de la Gloria, Naha, Masalniha, San Antonio Lindavista, Antela, and Tziscao have a majority (or totality) of peasant population. Other communities in this group, like Lacanja-Chansayab, Zinacantan, or Bazom, while not predominantly peasant, have a completely indigenous population.

Our results also support the model including gender and origin (Table 3). In this model men and natives are more mycophilic than women and migrants, respectively (Figure 6b-c). In many studies, the transcendental role of women in the process of wild mushroom management is indicated [17,44]. However, many groups from the Maya region allocate the role of going to the mountain and/or the milpa (cultivated fields) to the men [16]. As far as origin is concerned, when people migrate to lands with different conditions to their place of origin, pattern of species consumption are transformed, and traditional knowledge is displaced by global knowledge [20].

On the other hand, it is important to distinguish the description from the analysis of these patterns and their causes. In the historical process of settlement from highlands to lowlands in the Mayan area, mushrooms may have not been displaced from a cultural niche by plants as Mapes et al. [12] propose. Pre-Columbian Mayans did not become mycophobic, but rather maintained mycophilic attitudes and simply reformulated their knowledge and practices when introduced to the new resource that were tropical mushrooms.

However, evidence also shows cultural shifts in the region to be a product of urbanization and consequent separation from the environment, the abandonment of the milpa as the axis of productive life, the acquisition of global knowledge, and a transition in dietary habits [41]. These factors cause people to develop less mycophilic attitudes. The change linked to current events, such as lethal intoxications and the previously mentioned governmental actions and public policies, have generated fear and, consequently, mycophobic attitudes.

## Final considerations

While this study aims to help clarify the relations between people and mushrooms inhabiting different environments, as well as the causes of varying attitudes and practices, some questions remain: what is the situation in the rest of the Mesoamerica and other tropical regions? How are the frequency distributions among European peoples who were originally described as mycophiles or mycophobes under this model? Is their attitude towards mushrooms really dichotomic and exclusive, or rather a gradient, as seen in Chiapas? How are the attitudes among other Mesoamerican peoples who regard mushrooms as a highly important resource? While there are no other examples of systematic evaluations of mycophilia and mycophobia in different regions of the world, certain indicators suggest that other people might have a bell-shaped frequencies distribution. Fericgla [5] shows that, in Catalonia – a people recognized as lovers of mushroom consumption– there is no clear bias to mycophilia based on some quantitative data of practices related to local mycological knowledge. On the other hand, for peoples traditionally known for a scarce consumption of fungi species (e.g. peoples from the Gulf of Mexico) [43], there is no reason to think a frequencies distribution tending to mycophobia would be present. Additionally, worldview aspects must be further explored since they doubtlessly influence the manner in which peoples approach their natural resources.

Ethnomycology must keep looking for the most precise way to describe the attitudes among different peoples toward mushrooms, and proposing more complete and testable explanations to the observed reality.

## Abbreviations

AIC: Akaike Information Criterion; MMI: Mycophilia-Mycophobia Index; NTSYS: Numerical Taxonomy and Multivariate Analysis System; PCO: Principal Coordinates Analysis.

## Competing interests

The authors declare that they have no competing interests.

## Authors’ contributions

FRS designed the research study, did the fieldwork, and wrote early drafts of the manuscript. JCa reviewed and improved the design of the research and the manuscript, and collaborated in data analyses. CM participated in the revision of the research design and the manuscript and performed statistical analyses. JCi also reviewed both the design of the research and the manuscript; he also collaborated on everything relating to fungal species. ARG reviewed the design of the research and the manuscript. RGO collaborated in the first design of the research, as well as in data analyses and the revision of the manuscript. All authors read and approved the final manuscript.

## Supplementary Material

Additional file 1 Interview form used in the communities visited during study. Click here for file
