# Supplementary material for: Evaluation of the degree of mycophilia-mycophobia among highland and lowland inhabitants from Chiapas, Mexico
Source: J Ethnobiol Ethnomed. 2013 May 26;9:36. doi: 10.1186/1746-4269-9-36 (PMC3735042; doi:10.1186/1746-4269-9-36)
Supplement: Additional file 1 — Interview form used in the communities visited during study. [file 1746-4269-9-36-S1.pdf]

Appendix 1.

a) Piso ecológico:	b) Poblado:	c) ID de unidad familiar:

d) Nombre: g) Lengua materna:

e) Ocupación del padre de familia: h) Grupo de edad:

f) Procedencia:

**Preguntas**	**1**	**0.5**	**0**
1.- ¿Conoce usted hongos de monte? (I 1.1)	Si		No
2.- ¿Conoce los nombres de los hongos que se comen? (I 1.2)	Si		No
3.- ¿Usted o alguien de su familia trae hongos del monte para la casa? (I 1.3)	Si	Antes si, ahora no	No
4.- ¿Usted ha comido hongos del monte? (I 1.4)	Si		No
5.- ¿Le gusta comer hongos? (I 1.5)	Si		No
6.- ¿Los hongos son una comida especial, que le guste mucho? (I 1.6)	Si		No
7.- ¿Sabe cómo se cocinan? (I 1.7)	Una o más		No sabe
8.- ¿Qué siente usted cuando va al monte y no encuentra hongos que se comen en el monte? (I 1.8)	+	Ningún sentimiento particular	-
9.- ¿Existen hongos que hagan daño? (I 2.1)	Si		No
10.- ¿Cómo sabe que hongos hacen daño? (I 2.2)	+ Presencia de con.		- Ausencia de con.
11.- ¿Le preocupa o le da miedo tocar hongos que no conoce? (I 3.1)	No		Si
12.- ¿Usted ha escuchado si existen refranes o cuentos sobre los hongos? (I 4.1)	+	No se	-
13.- ¿Sabe usted si los hongos tienen otro uso, sirven para otra cosa? (I 5.1)	Otras cosas	No se	Nada
14.- ¿Conoce usted si hay personas aquí en la comunidad que sepan mucho de hongos y que vayan a buscarlos seguido? (I 6.1)	Si	No se	No
15.- ¿Qué hacen los hongos en el monte? ¿Para qué le sirven al bosque los hongos? (I 7.1)	+	No se	-
16.- ¿Hay animales que se acerquen a los hongos? ¿Cuáles y por qué? (I 7.2)	Si	No se / no salgo al monte	No
17.- ¿Ha visto si los niños en la comunidad o sus niños saben de hongos?, ¿les permiten que los agarren, jueguen con ellos o los lleven a la casa? (I 8.1)	Si	No se	No
18.- ¿Qué siente usted cuando va al monte y encuentra muchos hongos? (I 9.1)	+	Ningún sentimiento particular	-
19.- ¿Los hongos son importantes? ¿Por qué? (I 9.2)	Si	No se	No
